# Genome-Wide Analysis of the *KLF Gene* Family in Chicken: Characterization and Expression Profile

**DOI:** 10.3390/ani13091429

**Published:** 2023-04-22

**Authors:** Xuanze Ling, Qifan Wang, Jin Zhang, Genxi Zhang

**Affiliations:** 1 College of Animal Science and Technology, Yangzhou University, Yangzhou 225000, China; 2 Joint International Research Laboratory of Agriculture & Agri-Product Safety, Yangzhou University, Yangzhou 225000, China

**Keywords:** chicken, *KLF*, genome-wide identification, expression patterns, muscle, fat

## Abstract

**Simple Summary:**

Broilers provide nutrition for people all around the world, and their muscle and fat contribute major roles. However, little is known about the relationships between the kruppel-like factor (*KLF*) and the development of muscle and fat. In recent years, studies have shown that *KLF*s are involved in many physiological processes such as cell development, adipocyte differentiation and neurodevelopment. In our study, we aim to learn the functions *KLF*s play in the muscle and fat development of chickens. Various bioinformatics analyses were used to illustrate the genomic information of *KLF*s. A qPCR was performed to show the relative expression level of *KLF*s. In addition, we collected RNA-seq data and we found that *KLF*s have different expression levels in the same tissues at different points in time. This study helps explore the regulation mechanism of *KLF*s in skeletal muscle and fat, and provides a theoretical basis for broiler breeding.

**Abstract:**

The kruppel-like factor (*KLF*) gene family is a group of transcription factors containing highly conserved zinc-finger motifs, which play a crucial role in cell proliferation and differentiation. Chicken has been widely used as a model animal for analyzing gene function, however, little is known about the function of the *KLF* gene family in chickens. In this study, we performed genome-wide studies of chicken *KLF* genes and analyzed their biological and expression characteristics. We identified 13 *KLF* genes from chickens. Our phylogenetic, motif, and conserved domain analyses indicate that the *KLF* gene family has remained conserved through evolution. Synteny analysis showed the collinear relationship among *KLF*s, which indicated that they had related biomolecular functions. Interaction network analysis revealed that *KLF*s worked with 20 genes in biological processes. Kyoto Encyclopedia of Genes and Genomes (KEGG) pathway analysis showed that *KLF2* was involved in Apelin and Forkhead Box O (FOXO) signaling pathways. Moreover, qPCR showed that 13 *KLF* genes were expressed in the nine selected tissues and displayed various gene expression patterns in chickens. RNA-seq showed that *KLF3* and *KLF10* genes were differentially expressed in the normal and high-fat diet fed groups, and *KLF4*, *KLF5*, *KLF6*, *KLF7*, *KLF9*, *KLF12*, and *KLF13* genes were differentially expressed between undifferentiated and differentiated chicken preadipocytes. Besides, RNA-seq also showed that *KLF* genes displayed different expression patterns in muscle at 11 and 16 embryonic days old, and in 1-day-old chickens. These results indicated that the *KLF* genes were involved in the development of muscle and fat in chickens. Our findings provide some valuable reference points for the subsequent study of the function of *KLF* genes.

## 1. Introduction

Chicken meat is a good source of protein, with lower fat and cholesterol than other kinds of meats [1]. Ensuring the stability of chicken production is crucial in promoting socio-economic development in the face of population growth. Biological and pathological processes and other genetic factors are the main limiting factors affecting chicken growth and development [2]. In recent years, Molecular breeding for synchronized improvement of both meat yield and quality has become a popular topic of research on chicken [3]. Skeletal muscle plays an important role in locomotion, metabolism, and meat production in farm animals [4]. Many scholars have attempted to use the method of selective breeding to improve the chicken muscle growth rate [5,6,7]. A previous study showed that *KLF*s could control Fibroblast Growth Factor Receptor 1 (FGFR1) gene expression to regulate myoblast proliferation and differentiation [8].

*KLF* genes are characterized by three conserved zinc fingers in the DNA-binding domain, wherein the binding efficiency and transcription regulation can be affected by mutations [9]. Recent studies have revealed that *KLF* genes play diverse and essential roles in controlling metabolism at the cellular, tissue, and systemic levels. Up to now, 18 *KLF* genes have been identified in humans, named *KLF1* to *KLF18*. Moreover, *KLF* genes play various roles in different species such as mice, humans, pigs, etc. For example, *KLF1* acts as an essential transcription factor for erythroid development in mice [10]. *KLF1* is also proven to promote the proliferation, migration, and invasion of human lens epithelial cells by enhancing the expression of *ZBTB7A* (Zinc Finger and BTB Domain Containing 7A) gene [11]. Moreover, *KLF1* is involved in the growth and metabolism of pig muscle [12].

*KLF2* was found to regulate osteoblast differentiation by targeting *Runx2* in mice and humans [13]. *KLF3* is associated with the growth and development of muscle and adipose tissue in cattle [14]. *KLF6* is targeted by miR-152 to inhibit bovine myoblast proliferation [15]. *KLF4*, *KLF5*, *KLF6*, *KLF7*, *KLF10*, and *KLF15* are involved in the growth and development of mouse skeletal muscle [16,17,18,19,20,21]. The overexpression of *KLF9* in the mouse liver markedly increased blood glucose levels and impaired glucose tolerance [22]. *KLF12* was proven to regulate mouse Natural Killer cell proliferation [23]. *KLF8*, *KLF11*, and *KLF13* were demonstrated to be involved in human diseases [24,25,26]. Yang et al. [27] discovered that *KLF14* deletion resulted in increased fat mass in female mice and decreased fat mass in male mice. Knockout of *KLF16* could reduce oxidative stress and inflammation in mice hearts [28]. Wang et al. [29] found that *KLF17* may be critical for early mouse embryonic development. Up to now, no report was found regarding the function of *KLF18* in any animal.

The *KLF* gene family has been systematically identified in several animals, including pigs, mice, and cattle. However, the *KLF* gene family in chickens has not been thoroughly researched. In this study, we determined *KLF*s in chickens by building a HMM model and running a BLAST according to the available genome information. Phylogenetic analysis, gene structures analysis, motif, and conserved domain analysis were performed to understand the evolution and structural features of *KLF*s. Synteny analysis and interaction network analysis were performed to show collinear relationships and biological functions of *KLF* genes in chickens. Additionally, we used the Go enrichment and KEGG pathway analysis to explore the biological characteristics of *KLF* gene family. RNA-seq data was collected to show various gene expression patterns of the *KLF* genes in chickens. Furthermore, the expression patterns of *KLF*s in chickens were systematically investigated by qPCR analysis. This study would provide the fundamental basis for further functional studies of *KLF* genes.

## 2. Materials and Methods

### 2.1. Ethics Statement

This study is fully compliant with the codes made by the Chinese Ministry of Agriculture. The animal experiments performed in the study were all evaluated and approved by the Animal Ethics Committee of Yangzhou University (202103298).

### 2.2. Genome-Wide Identification of KLF Family Members in Chickens

The protein sequences of the *KLF* genes from *Homo Sapiens* (*H. sapiens*), *Mus musculus* (*M. musculus*), and *Sus scrofa* (*S. scrofa*) were retrieved and downloaded from the National Center for Biotechnology (NCBI) database. Then these sequences were compared with the protein sequences of *Gallus gallus* (*G. gallus)* using BLASTp with the expected value (E-value) of 1e ^− 10^ [30]. The *KLF* protein sequences were used as a query template to construct multiple alignment models using hidden Markov model software (HMM) to identify putative *KLF* genes in chickens using the presence of the zf-H2C2 domain [31].

### 2.3. Protein Alignment, Phylogenetic Analysis, and Chromosome Location Analysis of KLF Genes

To understand the evolutionary relationship of the *KLF* genes from these four species, multiple sequence alignments of identified *KLF* genes were performed using MUSCLE analysis [32]. The neighbor-joining (NJ) tree was constructed by Molecular Evolutionary Genetics Analysis (MEGA) 7.0 software with pairwise deletion of bootstrap value 1000 [33]. Conserved domains of *KLF* proteins were identified using CDD method in Batch software [34]. The SMART software also detected those domains found by a HMM model to confirm the final results [35]. The chromosomal locations of these *KLF* genes were derived from the genome annotation files downloaded from the Ensembl database [36]. Furthermore, the chromosome location of the *KLF* genes was displayed by TBtools [37].

### 2.4. Motif Identification and Conserved Domain Analysis of KLF Genes

The motifs of the *KLF* protein domain were identified using the Multiple EM (Expectation Maximization) for Motif Elicitation (MEME) program using the default parameters [38]. The number of motifs was set to 15. The minimum motif and maximum motif lengths were 6 and 50, respectively. The conserved domains of KLFs were searched in the Conserved Domain Database and displayed with TBtools [37].

### 2.5. Synteny Analysis and Interaction Network Analysis of KLF Genes in Chickens

The syntenic relationships between the *KLF* genes and gene density of *KLF* genes were analyzed by using TBtools. The interaction networks of chicken *KLF* genes were identified using the STRING database and the predicted interaction network was optimized by Cytoscape software [39,40].

### 2.6. GO Enrichment Analysis and KEGG Pathway Analysis of KLF Genes in Chicken

Gene Ontology enrichment analysis was used to identify the putative biological processes, cellular components, and molecular functions of *KLF* genes in chickens [41]. KEGG enrichment analysis was performed by KOBAS software to identify the functions of *KLF* genes in signaling pathways [42].

### 2.7. RNA-seq Results Analysis of the G. gallus KLF Genes

The first RNA-Seq data of 40 samples of testis, liver, lung, brain, kidney, intestine, muscle, and heart were obtained from the GEO DataSets and its GEO accession was GSE133401. The second RNA-Seq data derived from 12 samples of livers and abdominal fat in dwarf broilers were also obtained from the GEO DataSets and its GEO accession was GSE129840. The broilers began to be fed with a normal or a high-fat diet at 1 week old and they were executed to get livers and abdominal fat for extracting total RNAs at 8 weeks old [43]. The heatmap was made based on the sequencing data by R software [44].

The RNA-seq data of thigh muscle in Xinghua chicken was collected from GEO dataset and its GEO accession was GSE91060. Chicken leg muscles of three different developmental stages (11-day-old embryos, 16-day-old embryos, and 1-day post-hatch) were used to assess lncRNA and mRNA expression during chicken skeletal muscle development [45]. The sequencing data of preadipocytes that had not differentiated and six-day differentiated preadipocytes were obtained with this study.

### 2.8. Animals, Tissue Collections, RNA Extraction, and Quantitative Real-Time PCR (qPCR)

Heying Black chickens used in this study were provided by Jiangsu Heying Poultry Group Co., Ltd. (Sheyang, China). After artificial insemination, the eggs were collected and hatched at 37 °C and 60% humidity. The experiment group contained four male chickens and they were euthanized at 18 embryonic days (the sex was determined by the development of the gonad). Thigh muscle, pectoral muscle, heart, lung, glandular stomach, hypothalamus, liver, spleen and abdominal fat were collected. Subsequently, these tissues were snap-frozen in liquid nitrogen, and stored at −80 °C for RNA extraction.

Total RNA samples were extracted with TRIzol. The quality, concentration, and integrity of RNA samples were tested by spectrophotometer (Thermo, Waltham, MA, USA). Then 1 ug RNA of each sample was reverse-transcribed to cDNA by using HiScript QRT SuperMix for qPCR (+gDNA wiper) (Vazyme, Nanjing, China). The forward primers and reverse primers of *KLF*s were designed by Primer Premier 5.0 (Table S1). These primers were synthesized by Sangon (Shanghai, China). The qPCR experiments were conducted on Applied Biosystems 7500 (ABI, Los Angeles, California, USA) using SYBR qPCR Master Mix (Vazyme, Nanjing, China), and β-actin was used as housekeeping gene [46], with three technical repetitions for each sample. The 2^−ΔΔCT^ method was used to calculate the relative expression of *KLF*s [47].

### 2.9. Statistics Analysis

Statistical analysis was performed by using SPSS18.0 software (SPSS Inc., Chicago, IL, USA). A one-way ANOVA was used for multiple-group comparison analysis. Duncan’s multiple range test was used to determine significance. Unpaired Student’s *t*-test was used for a two-group comparison analysis. The data were considered statistically significant when *p* less than 0.05.

## 3. Results

### 3.1. Identification and Phylogenetic Analysis of KLF Genes

**Figure 1:**
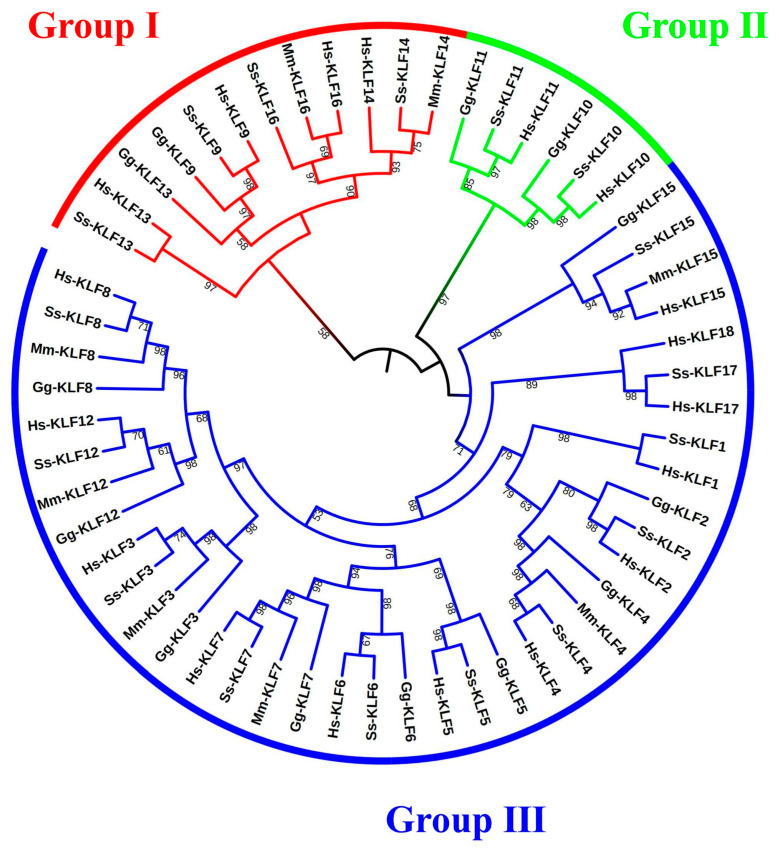
Phylogenetic analysis of *KLF* proteins in *G. gallus*, *M. musculus*, *S. scrofa,* and *H. sapiens*. The sequences of those 56 proteins were used to construct a neighbor-joining (NJ) tree. The tree was divided into three groups (I–III). “*H. sapiens*” was abbreviated to “Hs”. “*M. musculus*” was abbreviated to “Mm”. “*S. scrofa*” was abbreviated to “Ss”. “*G. gallus*” was abbreviated to “Gg”.

Based on the presence of the zf-H2C2 domain, we obtained 56 *KLF* genes in four species, including 18 *H. sapiens* genes, 8 *M. musculus* genes, 17 *S. scrofa* genes, and 13 *G. gallus* genes, respectively. To explore the evolutionary relationship among the members of the *KLF* families, an NJ tree based on protein sequences of these 56 genes from four species was constructed (Figure 1). Based on the alignment of the sequences and the phylogenetic analysis, 56 genes were divided into three groups, named groups I to III. Group I contained 12 *KLF*s, group II contained 6 *KLF*s, and group III contained 38 *KLF*s. We saw from the phylogenetic tree that *Ss-KLF13*, *Hs-KLF13*, *Gg-KLF13*, *Gg-KLF9*, *Ss-KLF9*, *Hs-KLF9*, *Ss-KLF16*, *Mm-KLF16*, *Hs-KLF16*, *Hs-KLF14*, *Ss-KLF14*, and *Mm-KLF14* are clustered into Group I. *Gg-KLF11*, *Ss-KLF11*, *Hs-KLF11*, *Gg-KLF10*, *Ss-KLF10*, and *Hs-KLF10* are clustered into Group II, and the remaining 38 *KLF*s are clustered into Group III.

### 3.2. Chromosomal Locations of G. gallus KLF Genes

**Figure 2:**
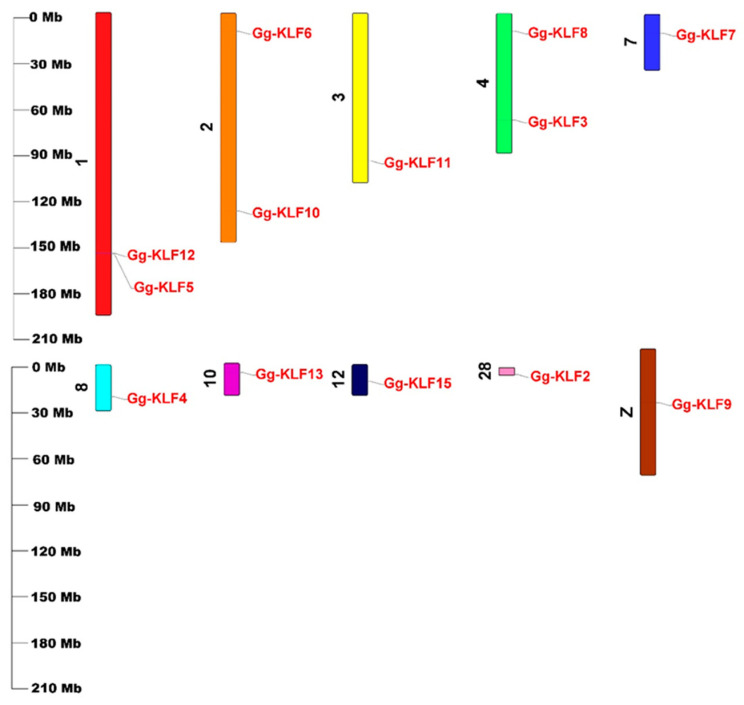
Chromosomal location of *KLF* genes on ten chromosomes in chickens.

The 13 *KLF* genes were distributed unevenly on ten *G. gallus* chromosomes (Figure 2). The accession numbers of the gene sequence, protein sequence, and coding sequence of the 13 *KLF* genes and their genomic locations were listed in Table 1. Chromosome 1 contained two genes, *Gg-KLF5* and *Gg-KLF12*. Chromosome 2 also had two genes, *Gg-KLF6* and *Gg-KLF10*, but these two genes were at different positions on the same chromosome. Like chromosome 2, chromosome 4 contained two genes, *Gg-KLF3* and *Gg-KLF8*. The remaining seven genes were distributed on the other seven chromosomes, meaning each had one *KLF* gene. *Gg-KLF11*, *Gg-KLF7*, *Gg-KLF13*, *Gg-KLF4*, *Gg-KLF2*, *Gg-KLF15*, and *Gg-KLF9* were found on chromosome 3, chromosome 7, chromosome 10, chromosome 8, chromosome 28, chromosome 12, and chromosome Z, respectively.

### 3.3. Motif Identification and Conserved Domain Analysis

**Figure 3:**
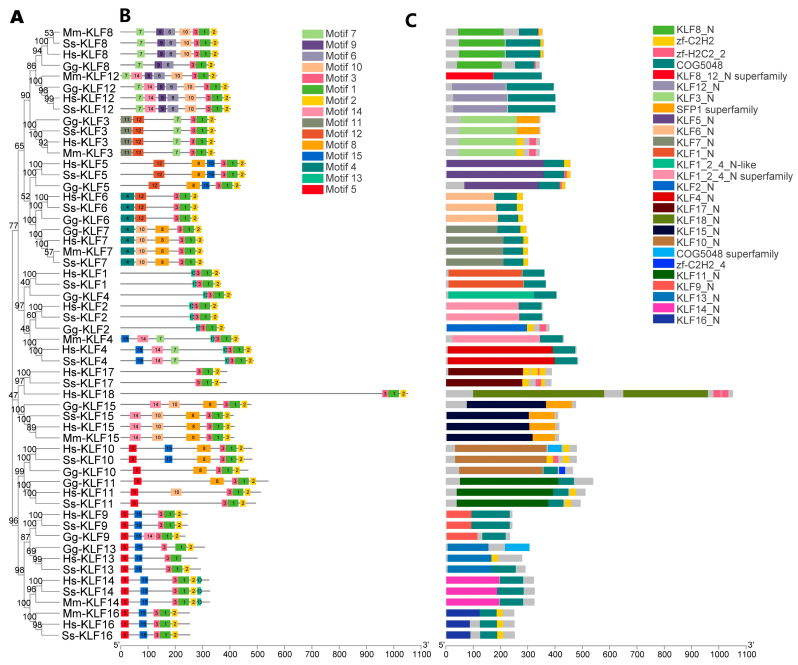
Phylogenetic relationships, Motif identification, and gene structure analysis. (**A**) Phylogenetic relationships of the 56 *KLF* genes identified in *G. gallus, S. scrofa*, *M. musculus*, and *H. sapiens* using the NJ method. (**B**) Motif analysis of the 56 *KLF* genes identified in *G. gallus*, *S. scrofa*, *M. musculus*, and *H. sapiens*. Colored boxes indicate conserved motifs and gray lines represent non-conserved sequences. (**C**) Conserved domain analysis of the 56 *KLF* genes identified in *G. gallus*, *S. scrofa, M. musculus*, and *H. sapiens*.

The phylogenetic analysis (Figure 3A) was combined with gene structures and motif analysis to indicate the relationship among *KLF* genes of *H. sapiens*, *M. musculus*, *S. scrofa*, and *G. gallus*. Using the HMMER program, we discovered that the *KLF* genes have 15 motifs. Motif 1 and motif 2 were present in all 56 *KLF* genes (Figure 3B). The most closely related genes in the same subfamilies shared common motif compositions, which may be indicative of similar functions. The conserved domain analysis illustrated that 27 conserved domains could be found in these 56 genes (Figure 3C). The genes clustered in the same group shared similar conserved domains.

### 3.4. Synteny Analysis and Interaction Network Analysis

**Figure 4:**
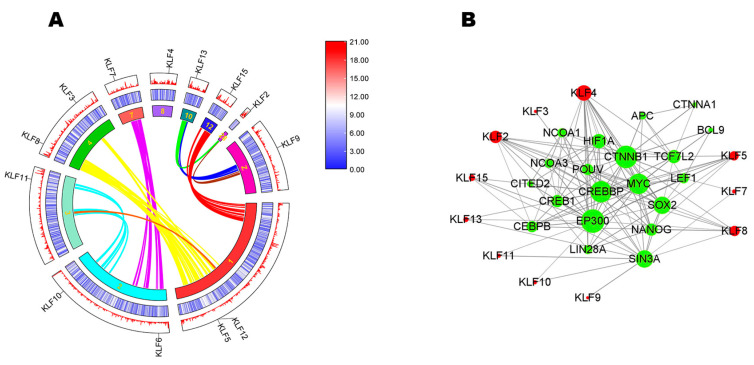
Synteny analysis and interaction network analysis. (**A**) The synteny analysis of *KLF* family in chicken. The innermost circle is composed of 10 chromosomes. The middle and outermost circles show gene density through a heatmap and line. The colors red, white, and blue represent high, medium, and low gene density, respectively. Gene names are shown at different locations of chromosomes. Curves of different colors represent the collinear relationship among different chromosomes and some of the *KLF*s had collinear relationships with each other. (**B**) Interaction network analysis. The interaction network shows the interaction relationship among *KLF*s and other genes.

In Figure 4A, the middle and outermost circles showed low gene density of all 13 *KLF*s in chicken. According to curves of different colors, we found that *KLF2* had a collinear relationship with *KLF13*. *KLF3* and *KLF8* had collinear relationships with *KLF5* and *KLF12*. *KLF6* had a collinear relationship with *KLF7*. *KLF10* had a collinear relationship with *KLF11*. Other *KLF*s didn’t have collinear relationships.

To better understand the biological function and the regulatory network of *KLF*s, their protein-protein interactions (PPI) were predicted (Figure 4B). The results showed that 11 *KLF*s had putative interaction relationships and 20 corresponding functional genes were found.

### 3.5. GO Enrichment Analysis and KEGG Pathway Analysis of KLF Genes in Chicken

**Figure 5:**
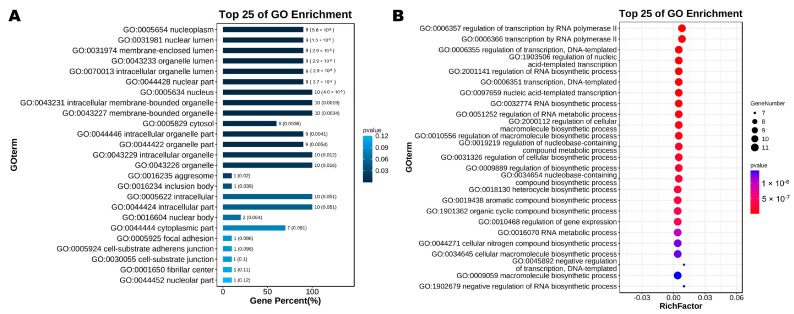
Go enrichment analysis of *KLF* genes in chicken. (**A**) The top 25 Go enrichment analysis of the cellular components of *KLF* genes in chicken; (**B**) The top 25 Go enrichment analysis of biological processes of *KLF* genes in chicken.

Figure 5A shows nine KLF proteins located at the nucleoplasm, nuclear lumen, membrane-enclosed lumen, organelle lumen, intracellular organelle lumen, and nuclear part, respectively, which were the top six GO Enrichment categories (Table S2). Figure 5B illustrates that 11 KLFs took part in biological processes like the regulation of transcription by RNA polymerase II, transcription by RNA polymerase II, regulation of gene expression, and so on (Table S3). According to the KEGG pathway analysis, we found only KLF2 was involved in Apelin signaling pathway and FOXO signaling pathway (Figure S1) [48]. Using KEGG, the 12 putative KLF proteins were not assigned to any signaling pathway.

### 3.6. Expression Profile Analysis of G. gallus KLF Genes

**Figure 6:**
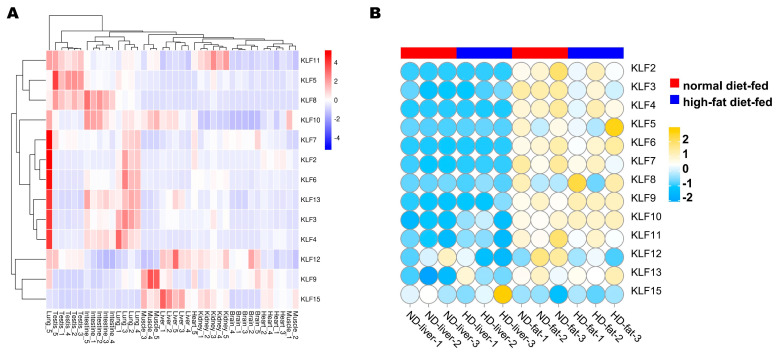
Expression patterns of the *KLF* gene family in chickens. (**A**) mRNA profiles of eight kinds of tissues from five adult roosters: testis, liver, lung, brain, kidney, intestine, muscle, and heart. Colors red, white, blue represent high expression, medium expression and low expression levels, respectively. (**B**) *KLF* genes in livers and abdominal fats between normal- and high-fat diet-fed dwarf broilers. The colors yellow, white, and blue represent high expression, medium expression, and low expression levels, respectively.

The *KLF* genes can be classified into four groups (Figure 6A) according to their differential expression patterns in tissues. Moreover, the eight chicken tissue types can also be clustered into eight main clades based on the expression patterns of all 13 genes. It could be seen from the expression profile that *KLF2*, *KLF3*, *KLF4*, *KLF6*, *KLF7*, and *KLF13* were expressed highly in the lungs. Further, *KLF9* was expressed highly in muscle. In Figure 6B, the expression levels of *KLF* genes were significantly higher in fat than liver, except for *KLF15*. The expression level of *KLF3* was significantly higher in the normal diet-fed group than the high-fat diet-fed group, while the expression level of *KLF10* was significantly lower in the normal diet-fed group than the high-fat diet-fed group.

### 3.7. QPCR Results of the G. gallus KLF Genes

**Figure 7:**
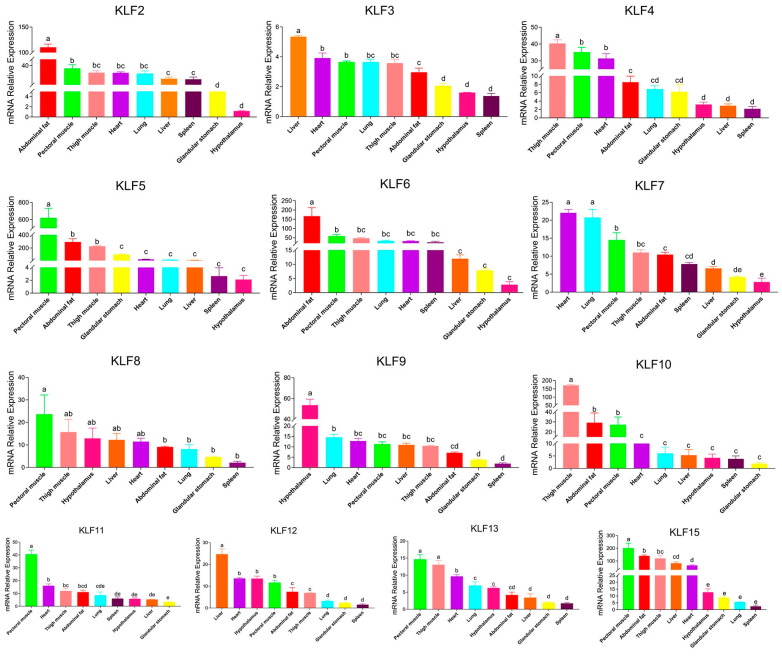
qPCR expression analysis of *KLF* genes in thigh muscle, pectoral muscle, heart, lung, glandular stomach, hypothalamus, liver, spleen and abdominal fat of four male chickens at 18 embryonic days old. Letters a, b, c, d, e and other lowercase letters denote a significant difference exists.

QPCR data was normalized with the same and unique normalizer gene, β-actin. The qPCR results showed that *KLF* genes had quite different expression patterns (Figure 7). The expression levels of *KLF2*, *KLF5*, *KLF6*, *KLF10* and *KLF15* were highest in thigh muscle, pectoral muscle and abdominal fat. *KLF4*, *KLF8*, and *KLF13* were most highly expressed in thigh muscle and pectoral muscle. We also discovered that the expression level of *KLF3* was highest in liver, followed by heart, pectoral muscle, lungs, thigh muscle, abdominal fat and other organs. The expression levels of *KLF7* were highest in the hearts and lungs, while the expression level of *KLF9* was highest in the hypothalamus. *KLF11* expressed highest in pectoral muscle but much lower in other organs. Like *KLF3*, the expression level of *KLF12* was highest in liver.

### 3.8. RNA-seq Result Analysis of the G. gallus KLF Genes

**Figure 8:**
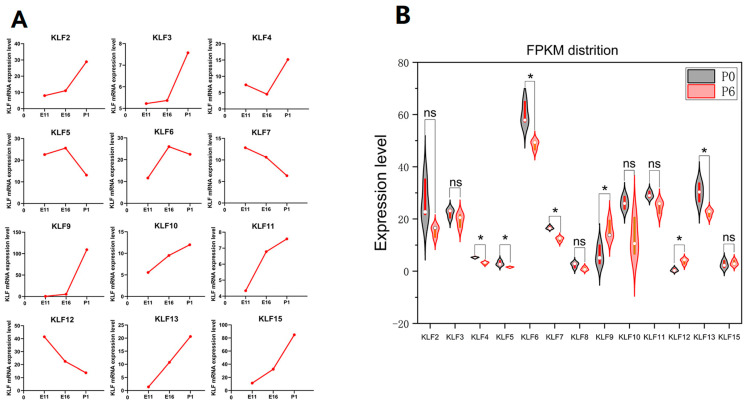
RNA-seq results analysis of the *G. gallus KLF* Genes. (**A**) mRNA relative expression levels of 12 *KLF* genes in leg muscles of Xinghua chickens at 11 and 16 embryonic days and 1 day old. (**B**) *KLF* mRNA relative expression levels in preadipocytes which had not differentiated and six-day-differentiated preadipocytes. P0 referred to preadipocytes which had not differentiated. P6 referred to six-day-differentiated preadipocytes. “ns” represents no significance. “*” represents *p* < 0.05.

The expression level of *KLF2*, *KLF3*, *KLF9*, *KLF10*, *KLF11*, *KLF13* and *KLF15* increased in samples from 11 embryonic-day-old to 1-day-old chicken (Figure 8A). The expression level of *KLF4* decreased between samples from 11 embryonic-day-old to 16 embryonic-day-old chicken, but it increased quickly from 16-embryonic-day-old to 1-day-old chicken. Contrary to *KLF4*, the expression levels of *KLF5* and *KLF6* rose between samples from 11 embryonic-day-old to 16 embryonic-day-old chickens, but it decreased quickly from 16-embryonic-day-old to 1-day-old chickens. The expression levels of *KLF7* and *KLF12* decreased between samples from 11 embryonic-day-old to 1-day-old chickens. As to *KLF8*, it was not included in the RNA-seq data and the expression of *KLF8* was absent. As Figure 8B showed, the expression levels of *KLF9* and *KLF12* increased significantly in differentiated preadipocytes, while the expression levels of *KLF4*, *KLF5*, *KLF6*, *KLF7*, and *KLF13* all decreased significantly after preadipocyte differentiation.

## 4. Discussion

It is known that *KLF*s are a family of transcription factors characterized by zinc-finger structures at the C-terminal end, and they play critical roles in cell proliferation, differentiation, and migration [49]. Using BLAST and HMM, we identified 56 *KLF* genes from four species and then a total of 13 *KLF* genes with specific conserved-domain zf-H2C2 genes were identified in *G. gallus*. The NJ tree was constructed with 56 gene sequences to compare chicken *KLF* genes with sequences from the three other species. The results demonstrated that the 56 *KLF* genes were divided into three groups. Comparative analysis suggested that chickens, mice, and humans have similar numbers of gene families [50]. Gene structure is an important factor in the evolution of gene families [51]. We found that the *KLF* gene structure of chickens is quite similar to that of humans, pigs, and mice. Motif analysis revealed that *KLF*s clustered in groups based on the similarity of their subdomains. The results of conserved-domain analysis showed that *KLF* genes are relatively conserved among these species. Among 20 genes predicted by interaction network analysis, *NCOA3* was found to participate in regulating porcine skeletal muscle satellite cell proliferation [52]. Furthermore, differential expression of *NCOA3* was proven to be regulated by miR-17-5p in pig intramuscular and subcutaneous adipose tissues [53].

Excessive fat deposition is considered an undesirable factor which affects feed efficiency, meat production costs, meat quality, and consumer health [54]. Hence, studies have been done to explore the relationship between *KLF* genes and fat deposition. A previous experiment proved that *Gg-KLF2* was highly expressed in abdominal adipose tissue, and its transcripts fluctuated during adipose tissue development [55]. Our research showed that the relative expression level of *KLF2* was highest in abdominal fat in male chickens. Genetic variants in the promoter region of the *KLF3* gene were associated with fat deposition in Qinchuan cattle [56]. *KLF3* was also demonstrated to be negatively correlated with intramuscular fat content in the longissimus dorsi muscle of Erhualian pigs [57]. The expression profile analysis found that *KLF3* might play important roles in the fat development process. In our research, the expression level of *KLF3* in abdominal fat was higher than that in the glandular stomach, hypothalamus, and spleen. However, there was no significant difference between the expression level of *KLF3* in differentiated and undifferentiated preadipocytes. Sometimes, *KLF* genes work together to produce a marked effect. Guo et al. found the *KLF15* gene was overexpressed through adenoviral vector (Ad-KLF15) in bovine adipocytes, and the expression level of the *KLF3* gene was increased, which indicated that *KLF15* promoted the transcription of the *KLF3* gene in bovine adipocytes [58]. Our synteny analysis showed that *KLF3* had collinear relationships with *KLF5* and *KLF12*, while interaction network analysis showed that 11 *KLF* genes had interaction relationships, including *KLF3* and *KLF15*. Besides, our research showed that *KLF15* was expressed at relatively high levels in abdominal fat. *KLF4* was proven to be one of potential therapeutic targets for obesity-induced cardiac injuries [59]. There was a significant difference between the expression level of *KLF4* in differentiated and undifferentiated preadipocytes in our research. Cardiomyocyte-specific *KLF5* knockout mice were found to have accelerated diet-induced obesity associated with increased white adipose tissue [60]. A qPCR showed that the expression level of *KLF5* was second highest in abdominal fat. The expression levels of *KLF5* decreased significantly after preadipocyte differentiation.

Iqbal et al. discovered that *KLF6* regulated fat synthesis in bovine mammary epithelial cells (BMECs) by targeting the *PPARA*, the *PPARG* pathway, and other fat marker genes [61]. Our research showed that *KLF6* expression was highest in abdominal fat. In our study, a significant difference was found between undifferentiated and differentiated adipocytes. RNA-seq result revealed that the down-regulation of the *KLF6* gene significantly up-regulated the genes that regulate adipogenesis, differentiation and regulation of adipocytes, and the homeostasis of bovine adipocytes, which proved that *KLF6* has a role in regulating lipid metabolism in bovine adipocytes [62]. *KLF7* was revealed to modulate the differentiation and proliferation of chicken preadipocytes [63]. We also found the expression level of *KLF7* significantly decreased after differentiation. *KLF8* was targeted by miR-10a-5p to inhibit the differentiation of goat intramuscular preadipocytes [64]. In our study, the expression level of *KLF8* decreased after their differentiation in chicken preadipocytes. *KLF9* was found to inhibit chicken intramuscular preadipocyte differentiation [65]. However, the expression level of *KLF9* increased significantly after chicken preadipocyte differentiation. *KLF10*, *KLF11*, *KLF12*, and *KLF13* function as positive organizers by a variety of different mechanisms, such as crosstalk with C/EBP and PPARγ, to regulate adipogenesis and associated pathways in cattle [66]. The expression profile analysis revealed that *KLF10* might play important roles in the fat development process. Our research showed that the expression of *KLF10* in abdominal fat was the second-most abundant of all the tissues tested, while no significant difference was detected between the expression level of *KLF10* in differentiated and un-differentiated preadipocytes. There was also no significant difference between the expression level of *KLF11* in differentiated and undifferentiated preadipocytes. However, we found the expression level of *KLF12* increased significantly and the expression level of *KLF13* decreased significantly in chicken preadipocytes.

*KLF* genes play not only important roles in fat deposition but also have great effects on skeletal muscle development. Manoharan et al. [67] identified *KLF2* as a key regulator of myeloid cell functions in mouse skeletal muscle regeneration. In our study, *KLF2* expression level increased in samples from 11 embryonic-day-old chicks to 1-day-old chicks. Moreover, *KLF2* was found to be involved in the FOXO signaling pathway. This FOXO signaling pathway played an important role in the pathogenesis of skeletal muscle atrophy by regulating E3 ubiquitin ligases and some autophagy factors [68]. This indicated that *KLF2* played an important role in chickens’ skeletal muscle development. Zhang et al. [69] confirmed that miR-21-5p promoted the proliferation and differentiation of skeletal muscle satellite cells (SMSCs) by targeting *KLF3*. The increasing expression level of *KLF3* in 1-day-old chicks might prove the function of *KLF3* for promoting muscle development. The *KLF4* gene was found to significantly promote the proliferation and differentiation of chicken primary myoblasts (CPMs) [70]. The expression level of *KLF4* was extremely high in chicken thigh muscle and pectoral muscle in samples at 18 embryonic days old in our research. A study suggested that *KLF5* may regulate the atrophy of chicken skeletal muscle through the Wnt/β-catenin signaling pathway [71], while *KLF5* expression levels decreased when chickens were hatched in our study. These results meant that *KLF5* displayed a vital role in the development of chicken muscle.

*KLF6* was confirmed as a new potential target gene of miR-148a-3p, and miR-148a-3p impeded bovine myoblast cell proliferation and promoted apoptosis through the posttranscriptional down-regulation of *KLF6* [72]. This conclusion corresponded with the downtrend of *KLF6* expression level in samples from 16 embryonic-day-old to 1-day-old chicks in our study. *KLF6* was also found to play a crucial role in regulating cell differentiation, proliferation, and muscle development, so it could be used as a potential candidate marker gene for the improvement of the Qinchuan cattle breed [73]. There is no report on the relationship between muscle development and *KLF7* or *KLF8*. In our research, the results in Figure 8A also showed that *KLF7* and *KLF8* might have a relationship with the development of muscle. RNA-Seq exploration of meat quality in Spanish goats illustrated that *KLF9* was associated with the effects of stress on skeletal muscle proteins [74]. We also found that *KLF9* expression levels were very high in the thigh muscle of one-day-old chicken. *KLF10* was known to control numerous genes in many cell types that are involved in various key biological processes like differentiation, proliferation, and apoptosis [75]. *KLF10* was expressed highly in the thigh muscle of Heying Black chickens and the expression level increased in samples from 11-embryonic-day-old to 1-day-old chicken, indicating that *KLF10* was involved in chicken muscle proliferation and differentiation. Furthermore, the sequencing result was used to show that *KLF11* was related to muscle development [76]. In our experiment, we also found the expression level of *KLF11* was high in pectoral muscle and that *KLF11* was also related to chicken muscle development. According to the RNA-seq results from the embryonic days samples and 1-day-old samples (Figure 8A), *KLF12* and *KLF13* might have the opposite effect on muscle development in chickens. *KLF15* was found to be a key regulator of branched-chain amino acid metabolism in the skeletal muscle of fish (tilapia) [77]. Both the RNA-seq and qPCR results showed that the expression level of *KLF15* was high in muscle, and its expression increased in samples from 11 embryonic-day-old to 1-day-old chicken, which indicated that *KLF15* was involved in the development of chicken muscle.

## 5. Conclusions

In this study, we identified 13 *KLF* genes in *G. gallus*. Phylogenetic analysis showed that the *KLF* genes could be divided into three groups. Chromosomal locations showed that the 13 *Gg-KLF*s were distributed on ten different chromosomes. Motif analysis and conserved domain analysis showed *KLF*s shared similar conserved domains in four species. Synteny analysis and interaction network analysis illustrated the collinear relationships and the relationships among *KLF*s and 20 corresponding genes. KEGG pathway analysis predicted that *KLF2* was involved in the Apelin-signaling pathway and the FOXO-signaling pathway. Our expression profile analysis revealed that the *KLF* genes displayed diverse expression patterns in chickens. This study provides some evidence that the *KLF* gene family plays a critical role in the development of chicken muscle and fat, which may give some suggestions for future research in the *KLF* gene family.

## Figures and Tables

**Table 1:** *KLF* gene family information in chickens (*G. gallus*).

Gene Name	Gene ID	Protein ID	CDS ID	Chromosome	Gene Position	
					Start	End
Gg-KLF2	ENSGALG00000003939	ENSGALP00000006265	ENSGALT00000006275	Chr28	4484818	4487291
Gg-KLF3	ENSGALG00000041717	ENSGALP00000053488	ENSGALT00000090511	Chr4	69533898	69558360
Gg-KLF4	ENSGALG00000047208	ENSGALP00000065832	ENSGALT00000097875	Chr8	20767770	20771114
Gg-KLF5	ENSGALG00000016927	ENSGALP00000086539	ENSGALT00000124066	Chr1	157437529	157456371
Gg-KLF6	ENSGALG00000033178	ENSGALP00000049438	ENSGALT00000073174	Chr2	11821968	11830999
Gg-KLF7	ENSGALG00000008501	ENSGALP00000013833	ENSGALT00000013848	Chr7	12266259	12323221
Gg-KLF8	ENSGALG00000037361	ENSGALP00000068386	ENSGALT00000099301	Chr4	11462278	11469482
Gg-KLF9	ENSGALG00000027374	ENSGALP00000043265	ENSGALT00000043393	chrZ	35032072	35045242
Gg-KLF10	ENSGALG00000037913	ENSGALP00000055499	ENSGALT00000059886	Chr2	129099993	129104922
Gg-KLF11	ENSGALG00000016440	ENSGALP00000075856	ENSGALT00000118896	Chr3	96486867	96495981
Gg-KLF12	ENSGALG00000016926	ENSGALP00000067131	ENSGALT00000099648	Chr1	156971380	157226168
Gg-KLF13	ENSGALG00000026590	ENSGALP00000081341	ENSGALT00000120340	Chr10	6022200	6048656
Gg-KLF15	ENSGALG00000036133	ENSGALP00000094959	ENSGALT00000124262	Chr12	11023377	11040828

## Data Availability

The data presented in this study are available on request from the corresponding author.

## Supplementary Materials

The following supporting information can be downloaded at: https://www.mdpi.com/article/10.3390/ani13091429/s1, Figure S1: Apelin signaling pathway and FOXO signaling pathway; Table S1: The forward primers and reverse primers of KLFs designed by Primer Premier 5.0; Table S2: The Go enrichment analysis of the cellular components of *KLF* genes in chicken; Table S3: The Go enrichment analysis of biological processes of *KLF* genes in chicken.
