# Supplementary figures and images for: Genome-Wide Analysis of the KLF Gene Family in Chicken: Characterization and Expression Profile
Source: Animals (Basel). 2023 Apr 22;13(9):1429. doi: 10.3390/ani13091429 (PMC10177326; doi:10.3390/ani13091429)

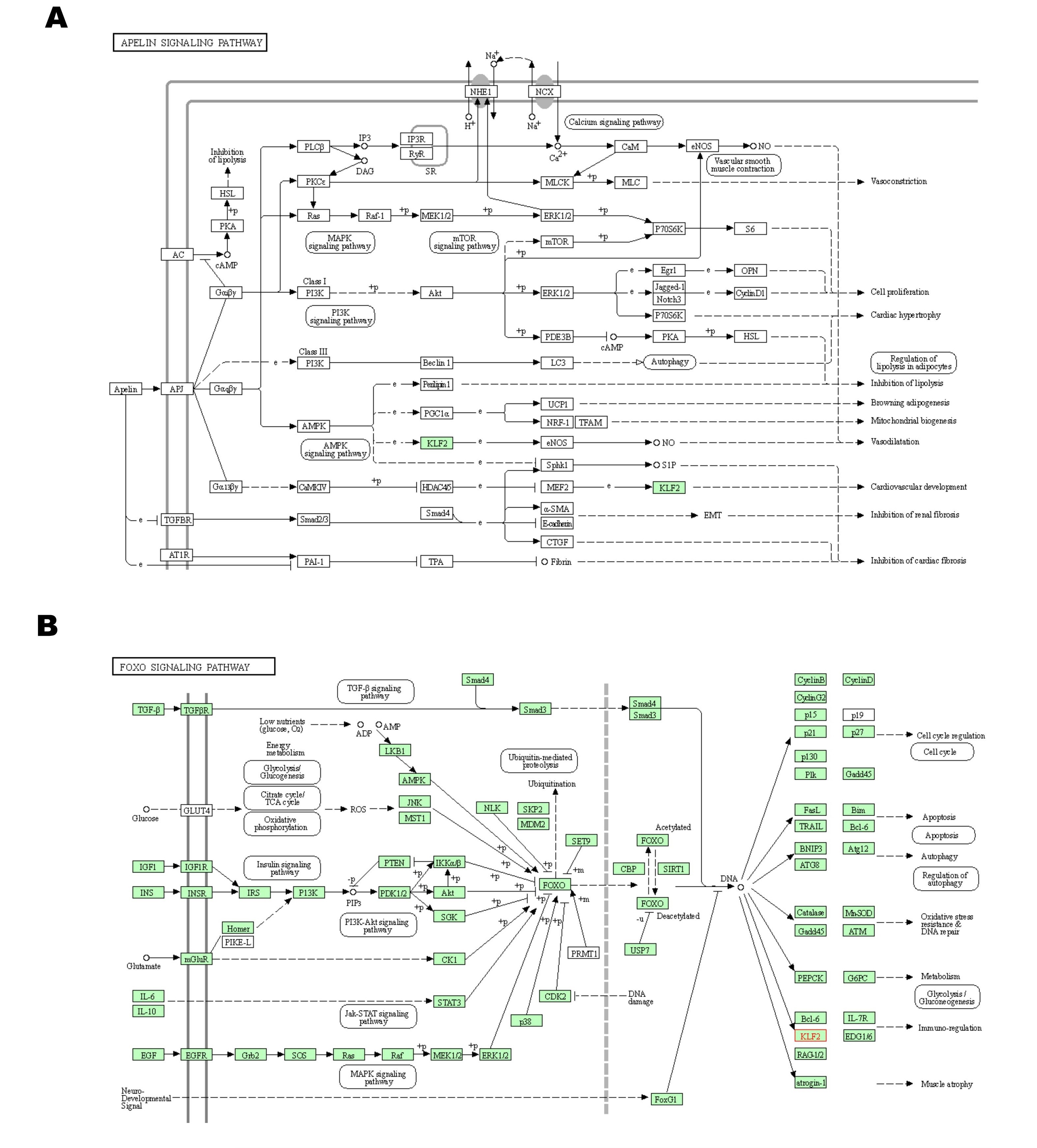

Supplement: Supplementary file 1 [file animals-13-01429-s001.zip › Figure S1.jpg]
